# Prevention of Aflatoxin Occurrence Using Nuts-Edible Coating of Ginger Oil Nanoemulsions and Investigate the Molecular Docking Strategy

**DOI:** 10.3390/plants11172228

**Published:** 2022-08-28

**Authors:** Amr Farouk, Adel Gabr Abdel-Razek, Karolina Gromadzka, Ahmed Noah Badr

**Affiliations:** 1 Flavor and Aroma Chemistry Department, National Research Center, Cairo 12622, Egypt; 2 Fats and Oils Department, National Research Center, Dokki, Cairo 12622, Egypt; 3 Chemistry Department, Poznan University of Life Science, ul. Wojska Polskiego 75, 60-625 Poznań, Poland; 4 Food Toxicology and Contaminants Department, National Research Center, Dokki, Cairo 12622, Egypt

**Keywords:** aflatoxins, antifungal activity, edible coating, enzymes responsible, ginger oil, molecular docking, nanoemulsion, nut seeds, shelf-life

## Abstract

The modern utilization of essential oils such as ginger oil (GO) as an anti-aflatoxin represents a potential target for food preservation and safety; however, the mechanism of action is still unclear. Nanoemulsions, through an edible coating, can enhance the oil’s bioactivity, increase its hydrophilicity, and extend the final product’s shelf-life. In the present study, two edible films for the GO nanoemulsion were prepared by ultrasonication using carboxymethyl cellulose (FB1-GO) and sodium alginate (FB2-GO). The droplet size of FB2-GO was finer (126.54 nm) compared to FB1-GO (289.77 nm). Meanwhile, both had high stability proved by z-potential; +31.54 mV (FB1-GO) and +46.25 mV (FB2-GO) with low PDI values (<0.4). Using gas chromatography-mass spectrometry, the hydrodistilled GO showed 25 compounds, representing 99.17% of the total oil, with *α*-zingiberene (29.8%), geranial (10.87%), β-bisabolene (8.19%), and *ar*-curcumene (5.96%) as the predominant. A dramatic increase in *α*-zingiberene, *α*-bisabolene and *ar*-curcumene was due to the homogenization conditions in both FB1-GO and FB2-GO compared to the GO. The FB1-GO exhibited superior antibacterial activity against the examined strains of bacterial pathogens, while FB2-GO was more effective as an antifungal agent on the tested *Aspergillus* fungi strains. In a simulated liquid media, FB2-GO inhibited the total growth of fungi by 84.87–92.51% and showed the highest reduction in the aflatoxin amount produced. The in silico study presented that, among the GO volatile constituents, sesquiterpenes had the highest binding free energies against the enzymes responsible for aflatoxin production compared to monoterpenes. α-Bisabolene showed the highest affinity toward polyketide synthase (−7.5 Kcal/mol), while *ar*-curcumene was the most potent against cytochrome P450 monooxygenase (−8.3 Kcal/mol). The above findings clarify the reasons for aflatoxin reduction in simulated media during incubation with FB1-GO and FB2-GO.

## 1. Introduction

The contamination of food commodities by toxigenic fungi and mycotoxins occurring during pre- and post-harvest has attracted the attention of scientific, political, and economic organizations [1,2]. Aflatoxins are harmful metabolites produced naturally under high humidity and moderate temperature conditions by toxigenic fungi, mainly created by *Aspergillus flavus* and *Aspergillus parasiticus* [3]. Aflatoxin is estimated to influence 25% or more of the world’s food crops annually [4]. Of all mycotoxins, aflatoxin B_1_ (AFB_1_) has been classified as a human carcinogen (Class I). It is reportedly the most toxic metabolite of toxigenic fungi [5]. Other aflatoxins, such as aflatoxin B_2_ (AFB_2_), aflatoxin G_1_ (AFG_1_), and aflatoxin G_2_ (AFG_2_), are all classified as potential human carcinogens (group 2) [6]. 

According to N’dede et al. [7], aflatoxins are a significant hazard affecting agricultural commodities, including cereals, peanuts, maize, yams, and almond. A wide range of ages consumes nuts as one of their diets. These foods provide vitamins, minerals, proteins, vital fatty acids for health, essential amino acids, and other nutrients. The daily intake of nuts was reported to be 8.9 g, which could vary significantly by country or location. It can be regarded as the average consumption of nuts based on nutrition surveys conducted worldwide [8]. For example, nuts or one type of nut make up around 15% of daily meals in European countries. Many nations currently use stringent rules for aflatoxins to promote the safety of nuts as high-risk contaminated food items. These requirements were primarily notable for the import and export of nuts [9].

A potential target for food producers and safety scientists is the control of fungal growth and the production of aflatoxins in food using environmentally friendly, biodegradable, and safer alternatives as opposed to synthetic chemicals that may be toxic to humans and have adverse environmental effects. Essential oils (EOs) are among the natural substances that have recently received a lot of interest as a potential strategy for reducing the generation of aflatoxins, particularly AFB1, in food. The consumers’ preference for natural food preservation techniques is the key argument for using natural products. Miri et al. [5] reviewed the anti-aflatoxigenic properties of EOs such as basil, cinnamon, cardamom, mint, cumin, coriander, rosemary, thyme, and many others. However, the mode of action of EOs has not been completely understood yet [5]. Direct uses of EOs may be restricted because of their poor dispersibility in hydrophilic fluids and sensitivity to environmental conditions. The EOs can be encapsulated in delivery systems to increase their solubility, stability, and regulated release and prolong their effectiveness over time. 

The creation of nanoemulsions increased the surface-to-volume ratio of these nano-sized delivery systems, improving their reactivity and enabling more efficient absorption into the cells and the controlled release and site-specific targeting of bioactive chemicals [10]. The emulsion of natural extracts was previously investigated to reduce mycotoxin contamination in food products. Several nanoemulsions were proven to minimize mycotoxin hazards, including *Nigella sativa* oil [11,12], by-product extracts [13], and piper oil [14]. Peanut, a highly contaminated nut with aflatoxins, was investigated for the preservation against aflatoxins using an edible coating such as glycerol [15] or Moringa oil and leaf extracts [16]. However, these materials are not considered EOs; their application in the nanoemulsion coating reflects significant results. The oil application in the crop coating to prevent aflatoxin contamination was successfully applied using jojoba oil to be a carrier for the bioactive components as a dried film [17] or a liquid film used such as a preserve solution [13], with high efficiency in inhibiting toxigenic fungi.

The preliminary information explains the motivation for this study, which also desired to formulate nanoemulsions containing the essential ginger oil (GO) and determine the anti-aflatoxigenic activity of these oil nanoemulsions via two edible films containing a carboxymethyl cellulose (CMC) base and alginate base. Following the previous survey, the physicochemical characteristics of the most potent nanoemulsion were studied. Parallel to the in vitro simulation of aflatoxin resistance using nanoemulsions, an in silico study through molecular docking was conducted with polyketide synthase and cytochrome P450 monooxygenase, which play a crucial role in AFB1 biosynthesis via the polyketide pathway (Caceres et al., 2020). The current study opens a perspective toward a better understanding of the anti-aflatoxin activity of ginger essential oil through an interpretation based on their chemical composition. It also employs compounds of botanical origin associated with nanotechnology to extend the shelf life of food products.

## 2. Results

### 2.1. Physiochemical Characterization of the GO Nanoemulsion

The mean droplet size of the FB1-GO and FB2-GO nanoemulsions and their PDI, z-potential, and stability index are shown in Table 1. The droplet size of FB2-GO (126.54 ± 8.41 nm) was more fine compared to FB1-GO (289.77 ± 15.34 nm). The emulsion composite size of the evaluated particle reflects the small-size droplets for film base 2 (FB2) more than the size of the average droplet for film base 1 (FB1). Nanotechnology’s capacity to create and optimize the unique physicochemical characteristics of microscopic composite structures is a crucial benefit. By modifying the size, shape, or surface chemistry of nanoparticles, their functionalities may be controlled by adjusting particular needs.

As shown in Table 1, the PDI of FB1-GO was recorded as 0.37 ± 0.05 and was decreased to 0.26 ± 0.07 for FB2-GO. The present study’s findings proved the efficiency of the ultrasonic technique during nanoencapsulation with a lower PDI value, which commonly means more emulsion stability, especially against creaming and sedimentation phenomena.

### 2.2. Ginger Oil and Its Nanoemulsion Composition (Nanoemulsion GC-MS)

Using GC-MS, 25 compounds representing 99.17% of the total oil content were identified (Table 2). The predominant compounds in the hydrodistilled oil were *α*-zingiberene (29.8%), geranial (10.87%), *β*-bisabolene (8.19%), *ar*-curcumene (5.96%), and *γ*-muurolene (5.21%). A dramatic change could be observed in the nanoemulsions compared to the HD oil, where ten compounds were identified in FB1-GO, accounting for 97.64% of the total volatiles. At the same time, only eight constituents were detected in FB2-GO, representing 99.96% of the whole extract (Table 1). The same aroma trend could be observed in the nanoemulsions compared to HD oil, where α-zingiberene, α- and β-bisabolene, and *ar*-curcumene were predominant, except for geranial and neral (Table 2). 

However, notable quantitative differences were observed due to the nanoencapsulation by HSH. α-Zingiberene showed a large increase in both FB1-GO (41.99%) and FB2-GO (39.96%) compared to the crude oil. Along the same line, α-bisabolene followed α-zingiberene with 14.96% for FB1-GO and 18.43% for FB2-GO, while *ar*-curcumene came as the third predominant with 14.52 and 16.23% for both FB1-GO and FB2-GO, respectively. Notably, the concentration of sesquiterpenes in the nanoemulsions was higher than in the GO. The previous findings seem to be related to the homogenization conditions and the amount of energy applied. It was noticed that the FB2-GO nanoemulsion was distinguished in sesquiterpenes and *ar*-curcumene. At the same time, *γ*-muurolene was recorded to be higher in the FB1-GO, which can explain its more anti-pathogenic effect on bacterial cells. 

### 2.3. Antibacterial and Anti-Aflatoxigenic Properties

The composites of two applied film materials were examined for their antimicrobial impact. The preliminary studies recorded the MIC as 370 to 450 µL/mL and 400 to 560 µL/mL for the FB1 and FB2, respectively. The values of MFC for the GO, FB1, and FB2 as raw composites were recorded at 0.017 to 0.021 mg/mL, 0.98 to 1.2 mg/mL, and 0.54 to 0.7 mg/mL, respectively. The results manifested the best antibacterial effect of the FB1 against the examined strains of bacterial pathogens, particularly after the GO was loaded into the particles (Table 3). The essential oil of ginger was noticed by its effective impact as an antimicrobial agent, particularly against toxigenic fungi. The ginger essential oil nanoemulsions would exhibit an antibacterial effect due to their specific active compound chemical structure. Otherwise, the composite of the FB2 recorded a more antifungal impact on the tested *Aspergillus* strains. These results are compatible with the results recorded of the raw composites for the MIC and the MFC values.

Compared to the GO activity, the FB1-GO was recorded to possess a more anti-pathogenic effect for the applied strains of bacteria. In contrast, an anti-mycotoxigenic fungal impact was noticed for the FB2-GO. The result against Gram-positive bacterial strains was more than the Gram-negative impact, which may connect to the cell membrane structure. The fungal strain of *A. parasiticus* ITEM 11 was the least sensitive strain among the investigated *Aspergillus* and was more resistant to all forms of GO applications. 

### 2.4. Anti-Aspergillus and Anti-Aflatoxin Simulation Experiment of Emulsion Composites

The film composites (FB1 and FB2) were applied in the simulated liquid media of two aflatoxigenic strains, and a considerable reduction in mycelial growth weight was recorded (Table 4). The antifungal effect of the FB2 was more apparent for the raw composite than that of the FB1 recorded. In the same line, the final inhibition ratio of the FB2 loaded by the GO ranged between 84.87 and 92.51% of the total mycelial growth of fungi. The antifungal impact of the applied material is expected to participate in the aflatoxin reduction ratio. According to the literature, the efficacy of the GO reduced the mycelial growth of *Aspergillus flavus* significantly at a concentration of 150 µg/mL, and complete inhibition of conidial germination was observed at a concentration of 10 µg/mL. The GO effect on mycelial fungal inhibition was recorded at 44.93% and 45.93% for the applied strains in liquid media of *A. flavus* and *A. parasiticus*, respectively. The ratios were enhanced for oil applied in FB1-GO form and had the best impact by oil application in the form of FB2-GO to the liquid media of fungal growth. The highest mycelial inhibition was recorded for the FB2-GO added to the liquid media containing *A. flavus* spores, and it was recorded as 92.51% inhibition. 

**Figure 1:**
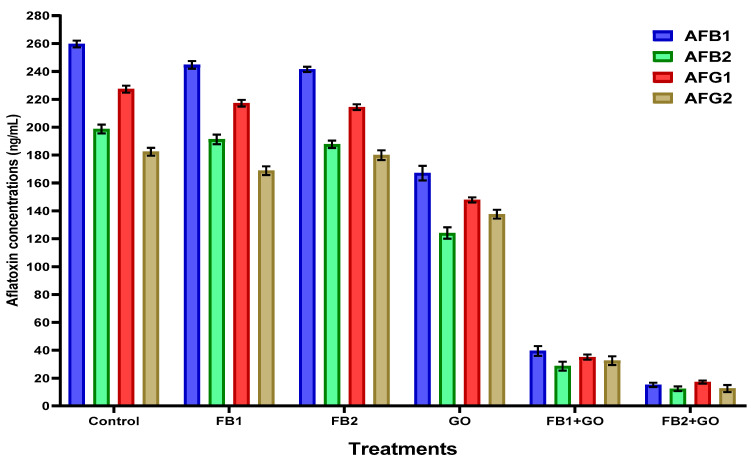
The applied composites (FB1, FB2, and GO-loaded emulsions) affected the aflatoxin production in the simulated liquid media. The results were represented as mean ± SEM (standard error means; n = 3). The results were expressed as aflatoxin reduction measured in nanogram/milliliter (ng/mL). FB1—coated emulsion composite consisted of maltodextrin, CMC, Arabic gum, and Guar gum. FB2:—coated emulsion composite consisting of sodium alginate, whey protein, and GO—ginger oil.

In the same liquid medium of the simulated experiment, aflatoxins of AFB_1_, AFB_2_, AFG_1_, and AFG_2_ were determined, and the degradation of aflatoxin production is shown in Figure 1. The aflatoxin quantities were recorded only for strain *A. flavus* ITEM 698, as it is known from previous investigations as a high aflatoxin-producing strain. The reduction in the aflatoxin amount was evident regarding the media growth containing the FB2 emulsion.

### 2.5. Evaluation of Molecular Docking

**Figure 2:**
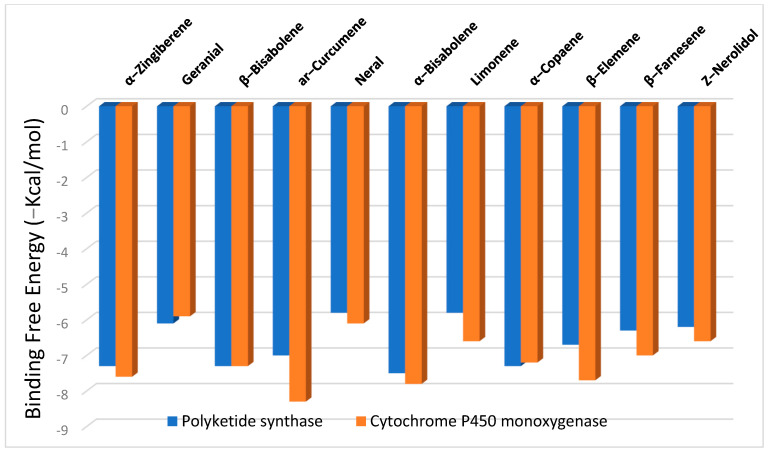
The binding free energy values were calculated by the molecular docking of significant constituents in ginger essential oil as ligands with polyketide synthase and cytochrome P450 monooxygenase.

The binding free energies (∆G) for the significant constituents of ginger essential oil as ligands with polyketide synthase and cytochrome P450 monooxygenase receptors are shown in Figure 2, revealing the best poses obtained in the molecular docking analyses. The lower the ∆G, the more significant the interaction between the receptor and the ligands with potential activity. Generally, sesquiterpenes displayed higher binding affinities than monoterpenes at both receptors, with high docking scores of −6.2 to −7.5 kcal/mol at the polyketide synthase and from −6.6 to −8.3 kcal/mol at the cytochrome P450 monooxygenase.

The findings above-mentioned may reveal the activity of the ginger oil constituents in suppressing the formation of aflatoxins (as observed in Table 2) due to the interaction with the main enzymes responsible for the mycotoxin formation. Among the sesquiterpenes, *α*-bisabolene showed the highest binding affinity toward polyketide synthase (−7.5 kcal/mol), while *ar*-curcumene was the most potent against cytochrome P450 monooxygenase (−8.3 kcal/mol).

**Figure 3:**
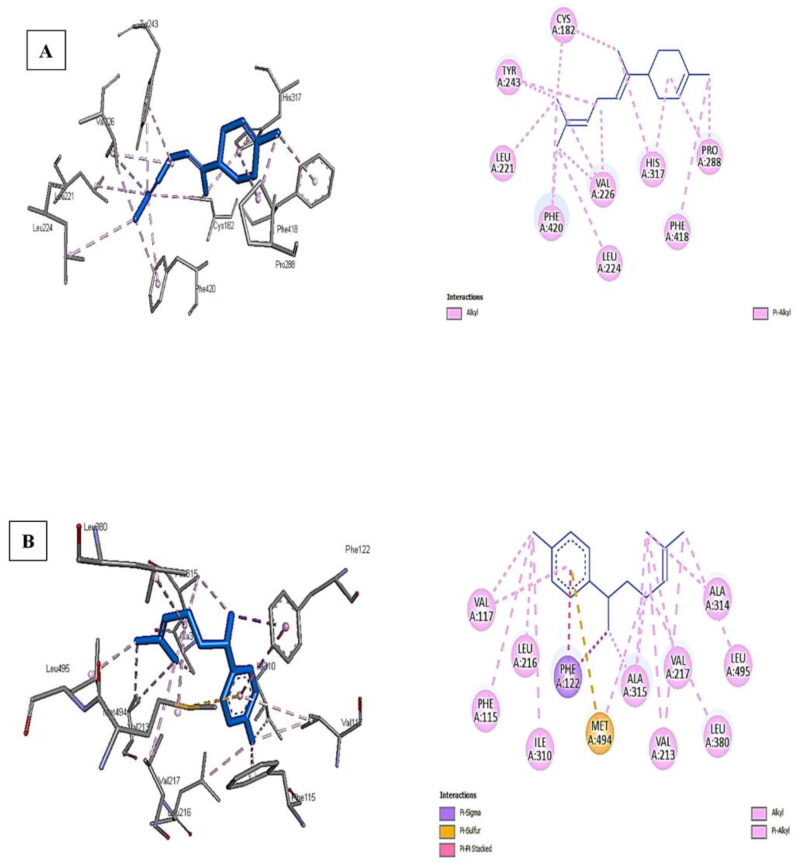
The interactions of *α*-bisabolene with polyketide synthase (**A**) and *ar*-curcumene with cytochrome P450 monooxygenase (**B**).

Figure 3A,B shows that the interaction of *α*-bisabolene with polyketide synthase and *ar*-curcumene with cytochrome P450 monooxygenase is the most potent among the best docking scores. The higher binding affinity of *ar*-curcumene with cytochrome P450 monooxygenase is attributed to the pi-sulfur bond formed with MET A:494, pi–pi stacked with PHE A:122, in addition to other bonds such as pi-sigma, alkyl, and pi-alkyl with many residues of the receptor-like VAL A:117, LEU A:216, PHE A:115, ALA A: 315, 316, VAL A: 217, 213, and LEU A:380,495 (Figure 3B). 

The conventional H-bonds were missing for both tested ligands against the receptors due to their chemical structure and lack of oxygen or more electronegative atoms. On the other hand, only alkyl and pi–alkyl bonds were observed between *α*-bisabolene and polyketide synthase moieties such as CYS A:182, TYR A:243, LEU A: 221, PHE A:420, and many others (Figure 3A). Again, the types of interactions and the number of specific bonds such as pi–sulfur were the main reasons for the differences in binding affinity between ligands against different receptors.

## 3. Discussion

The technique and conditions of nanoencapsulation and types of emulsifiers or biopolymers used extensively affect the droplet size, stability, and chemical structure of the nanoemulsions formed [18]. For example, ultrasonication of GO with Tween 80 led to nanoemulsions with a droplet size of 57.4 ± 2.7 nm [19]. This finding explains the obtained sizes of the oil nanoparticles in the present study and the emulsion stability. Many researchers, including Wu et al. [20] and Artiga-Artigas et al. [21], prepared nanoemulsions by incorporating biopolymer solutions such as sodium alginate and chitosan before the sonication process and reported droplet sizes of 169–490 nm due to the biopolymer aggregation and formation of a dense and thick layer on the surface of the oil droplets, which led to an increase in the diameter [19]. Tween 80 is favorable for oil-in-water emulsions among the different emulsifiers due to its higher hydrophilic-lipophilic balance (HLB-15). As a small molecule surfactant, it can minimize the droplet diameter compared to biopolymers due to its rapid adsorption onto the droplet surface [22].

The large negative or positive zeta potential avoids the tendency of repelling the particles to each other in the suspension by diminishing the probability of aggregation. This finding was clear for the stability of the formed emulsion, and high zeta-potential values were noticed. If the absolute value of the z-potential is between ±30 and ±60 mV, the nanoemulsion system is stable [23]. The FB2-GO nanoemulsion showed a larger z-potential (+46.25 ± 1.05 mV) and, therefore, more stability in comparison to FB1-GO (+31.54 ± 1.08 mV) (Table 1). Using biopolymers to prepare nanoemulsions showed higher z-potential values than applying emulsifiers such as Tween 80. For instance, using chitosan resulted in +57.33 mV [20], while applying sodium alginate produced −71 mV [24] stabilized EO nanoemulsions. In contrast, the preparation of nanoemulsions containing GO with Tween 80 resulted in a lower z-potential (18.70 ± 1.32 mV) [19]. Therefore, using biopolymers instead of low molecular weight surfactants increased the z-potential causes of more electrostatic repulsion, and more stabilization of the biopolymer emulsified nanoemulsions. The emulsion stability (ES %) data (Table 1) revealed that the nanoemulsions prepared were kinetically stable, especially for the coated systems containing sodium alginate and whey protein (FB2-GO) due to their excellent emulsifying capacity [25].

The above results in Table 2 revealed that the examined ginger belonged to the *α*-zingiberene chemotype (monocyclic sesquiterpene hydrocarbon), as reported for both the Egyptian and Chinese rhizome varieties by Ali et al. [26] and Al-Dhahli et al. [27]. However, some quantitative differences were observed as a result of endogenous and exogenous factors such as individual genetic variability, differences in growing locations, agronomical practices, and environmental conditions, which were reported as the primary reasons affecting the chemical composition of essential oils [28]. Numerous investigations have found that the main ingredient of ginger rhizomes from Ghana, Thailand, Poland, Nigeria, Australia, and India was *α*-zingiberene. In contrast, the predominant oil component from Saudi Arabia, Cuba, and Brazil was *ar*-curcumene [27,29,30]. It is noteworthy that other volatile constituents were detected as significant constituents in the present investigation in contrast to the study by Ali et al. [26], such as geranial (10.87%), neral (5.21%), and farnesene isomers (4.76%), which agreed with Nerilo et al. [29], who reported the same in the Brazilian cultivar.

Most studies on the encapsulation of flavors or oils concentrate on the physical stability and biological activity of micro- or nanoparticles rather than potential alterations to the volatile components of the encapsulated oils. For instance, Ali et al. [18] observed that the anticancer and antioxidant activity of the nanoemulsions differed from the original HD oil. The formulation based on energy-intensive procedures such as ultrasonication, high-speed homogenization, and high-pressure homogenization may result in Ostwald ripening, flocculation, or coalescence of the emulsion, with changes in its physical stability and biological activity, according to Aouf et al. [30].

The values of MFC for the FB1 and FB2 as raw composites were recorded at 0.98 to 1.2 mg/mL and 0.54 to 0.7 mg/mL, respectively (Table 3). The previous findings agreed with Ruan et al. [31], who reported high antimicrobial and oil barrier properties for edible coatings made of sodium alginate, and Azhdari and Moradi [32], who showed a significant decrease (*p* < 0.05) in all microbial groups (*Aspergillus flavus*, *A. fumigatus*, *A. niger*, *Penicillium citrinum*, and *Candida albicans*) upon the applied CMC coating in high-moisture mozzarella cheese. The ginger essential oil nanoemulsions would exhibit an antibacterial effect due to their specific active compound chemical structure. According to Firoozi et al. [33], the produced GO nanoemulsion using gum arabic and Tween 20 showed higher bactericidal activity against two common Gram-positive and Gram-negative bacteria, namely, *S. aureus* and *E. coli*, respectively. Previous research has confirmed the antibacterial activity of the micro-structure GO against Gram-negative and Gram-positive bacteria including *E. coli* and *S. aureus*. However, the higher MIC and MBC values for ginger essential oil for *S. aureus* than *E. coli* indicated that it had better antibacterial activity against Gram-negative bacteria, which was also supported by the findings of the current investigation for nano-sized essential oil [34]. The antimicrobial activity of GO against Gram-negative cells is related to their lipophilic structure, which may attach to the bacterial cell membrane and interfere with the penetration of routine compounds and destabilize their transport, leading to the collapse of the proton pump and ATP depletion, ultimately killing the cells. 

The effects of the extracted GO and its prepared nanoemulsion using Tween 80 only on the mycelia growth of *A. niger* during seven days of incubation (26 ± 2 °C) revealed that the control plate experienced rapid growth. In contrast, the plates containing GO and its prepared nanoemulsion significantly (*p* < 0.05) inhibited mycelial growth [35], which agreed with the findings of the present study. According to the obtained data, ginger (O/W) nanoemulsions demonstrated greater fungicidal efficacy than the pure ginger essential oil. It appears that the connected nanoemulsion interferes with the budding process of fungi, killing off the strains in the process. The results reflect the high efficiency of the FB2 to inhibit close to a quarter amount of mycelial growth. The previous finding is supported by Tøndervik et al. [36], who showed the ability of sodium alginate to inhibit the fungal cell growth of Candida and *Aspergillus* spp. In addition, GO could fully inhibit aflatoxin production by *A. flavus* at a concentration of 15 µg/mL [29]. The antifungal activity and inhibition of aflatoxin production for GO are well-reported in the literature due to its bioactive constituents, especially *α*-zingiberene (23.85%) and geranial (14.16%), in line with the results of the present study [26,29]. This impact could be elucidated through the release efficiency of this composite for the GO-loaded material during the incubation time of spores in the growth media. Additionally, the particle size and zeta-potential values could enhance the emulsion penetration for the fungal cell wall and affect the metabolic processes [37].

The current results may suggest the activity of ginger oil components in reducing the formation of aflatoxins, as shown in Table 2, related to interactions with the primary enzymes involved in mycotoxin formation. Among the sesquiterpenes, α-bisabolene had the most excellent affinity for a polyketide synthase (−7.5 kcal/mol), while *ar*-curcumene had the highest affinity for cytochrome P450 monooxygenase (−8.3 kcal/mol). Interestingly, Al-Dhahli et al. [27] discovered that sesquiterpenes in Saudi and Chinese ginger oils had a greater binding affinity than monoterpenes for inhibiting bacterial protein. Notably, all relevant compounds tested against both receptors yielded comparable molecular docking data. To our knowledge, nothing in the literature concerning the in silico studies involved inhibiting the key enzymes responsible for aflatoxin biosynthesis. Recently, a related study by Kumar [38] to inhibit the PT-domain of polyketide synthase A (PksA), which regulates aldol cyclization and aromatization to produce polyketide precursors, results in the creation of secondary metabolites (aflatoxin) utilizing the ginger phenolic extract. [6]-Gingerol and its derivatives such as (S)-[8]-gingerol, 9-Hydroxy-[6]-gingerol, 9-gingerol, 5-O-methyl-[9]-gingerol, methyl-6-gingerol, and [5]-gingerol exhibited better binding affinity, however, the author did not report any values in the study. Leu1508, Asn1554, and Asn1568 are the key amino acid residues involved in stabilizing ligand-protein interaction.

## 4. Materials and Methods

### 4.1. Materials

All of the chemicals applied in this investigation were of HPLC-grade and were purchased from Sigma-Aldrich, Saint Louis, MO, USA. The used strains of bacteria for antibacterial evaluation were *Listeria monocytogenes* ATCC 15313, *Bacillus cereus* NRRL 569, *Klebsiella aerogenes* ATCC 13048, and *Pseudomonas aeruginosa* ATCC 9027. These strains were obtained from the Egyptian Microbial Culture Collection (EMCC), Ain Shams, Egypt. However, toxigenic strains of fungi such as *Aspergillus flavus* ITEM 698, *A. parasiticus* ITEM 11, and *A. nomius* NRRL 13137 were utilized for antifungal evaluations. These fungal strains were obtained from the Agrofood Microbial Culture Collection (ITEM), ISPA, Bari, Italy. 

### 4.2. Methods

#### 4.2.1. Essential Oil Extraction by Hydro Distillation 

The experiment was performed in triplicate. The ginger rhizomes (*Zingiber officinale*) of the Egyptian plant were purchased from an identical herbal farm at Ismailia, Egypt (30.5965° N, 32.2715° E) after it was sundried and milled to a fine powder. Samples (*Zingiber officinale Roscoe*) (100 g) were subjected to hydrodistillation for three hours using a Clevenger-type apparatus, following the protocol of Farouk et al. [28]. The extracted essential oil was treated using anhydrous sodium sulfate and stored in airtight glass vials covered with aluminum foil at (−20 °C) until analysis.

#### 4.2.2. Preparation of Applied Film Composites

A novel edible film was formulated using food-grade materials to enhance the food product’s safety properties. Two composites were formed: film base (1) and film base (2). The emulsion of film base (1) was composed of maltodextrin, carboxy-methyl cellulose (CMC), gum arabic, and Guar gum. Each of the previous materials was prepared as a solution in double-distilled water. The applied powder concentration was 10% (*w*/*v*) maltodextrin, 3% gum arabic, Guar gum, and the CMC. Afterward, several quantities were utilized for preparation as follows: 250 mL maltodextrin + 500 mL CMC + 210 mL Guar gum + 40 mL gum arabic to form 1 L of emulsion. After well blending using magnetic stirring, glycerol and Tween 80 were added as 1% of the total volume to enhance the emulsification and softness characteristics, and 1% *w*/*v* GO as an anti-aflatoxigenic material.

Whey protein isolates as an encapsulating agent at an optimum concentration were applied to obtain stable emulsions (5% *w*/*v*). With modifications, the film base (2) was prepared according to Riquelme et al. [39]. The active film formed as an emulsion containing 1% *w*/*v* sodium alginate as a matrix and whey protein (10%; *v*/*v*). The film was created using a mix of these two solutions (1:1; *v*/*v*), then 1% (*v*/*v*) of sorbitol was added to the mixture as a plasticizer. After well blending for one hour by a mechanical stirrer, glycerol and Tween 80 were inserted as 1% of the total volume to enhance the emulsification and softness characteristics, and 1% *w*/*v* GO as an anti-aflatoxigenic material. 

#### 4.2.3. Preparation of the GO Nanoemulsions

The coarse emulsion was prepared [24] by dissolving the oil in T80 using a magnetic stirrer (30 min). The oil-T80 mixture was added to emulsion (1% *v*/*v*) to prepare the coarse solution. The final solution was stirred for 1 h using a magnetic stirrer until complete homogenization. Film forming emulsions were prepared using mechanical stirring for 2 h at 1500 RPM followed by ultrasonication treatment according to the methodology and equipment described by Ultrasonic Microprocessor (VCX 500), Fisher Scientific, Bishop Meadow Rd, Loughborough LE11 5RE, UK. Ultrasound was used in a 500 W (20 kHz) Autotune High-Intensity Ultrasonic Processor. The probe was immersed in the oil emulsion solution. where the sample was sonicated for 10 min/20 kHz with 1 pulse/s and 30% acoustic power. The samples were iced to 4 °C during the ultrasonic treatment to avoid overheating. Active films were prepared by casting at 42 °C for 20 h from film-forming emulsions dried on the nut seeds to protect them against contamination.

#### 4.2.4. Characterization of the Nanoemulsions

The nanoemulsions were evaluated for characterization as the particle size (PSz), zeta potential (ZP), and poly dispersing index (PDI) as the same methodology described by Malik et al. [40]. The Malvern apparatus (Nano-S90, Zetasizer, Malvern Panalytical Ltd., Enigma Business Park, Grovewood Road, WR14 1XZ, UK) was utilized to estimate the PSz, ZP, and PDI values. The emulsion was transferred in a 50 mL graduated bottle, capped, and stored for 5 days at 25 °C [41]. The serum separation from the emulsion will be taken as the emulsion stability index. The separated serum height of the emulsion surface monitored % stability is calculated using the equation:

%ES=(H1/H0)×100

where **ES** is the emulsion stability; **H1** is the upper phase height; H0 is the initial emulsion height.

#### 4.2.5. Gas Chromatography-Mass Spectrometry (GC-MS)

The essential oil constituents produced from HD were examined using the GC-MS instrument. A Thermo Scientific Trace GC Ultra Chromatography system equipped with a 60 m × 0.25 mm × 0.25 µm TG-5MS capillary column (Thermo Scientific, 168 3rd Ave, Waltham, MA 02451, USA) and ISQ-mass spectrometer were used for the separation. After isothermal holding at 50 °C for 3 min, the column temperature was raised by 4 °C per minute to 140 °C with a holding period of 5 min. Afterward, the temperature rose at a rate of 6 °C per minute before reaching 260 °C for an isothermal holding period of 5 min. The carrier gas was helium, with a constant flow rate of 1.0 mL/min. The injector temperature was 180 °C, the transition line was 250 °C, and the ion source was 200 °C. The mass spectrometer was configured with an ionization energy of 70 eV and a scan range of *m*/*z* 40–450. The MS computer library (NIST library, 2005 version), a comparison with standards, and published data were used to identify the compounds. The GC peak areas were used to compute the percentages of the detected elements. The retention times of a homologous series of C6–C26 n-alkanes were used to determine the Kovats index for each compound, and the values were compared to those found in the literature [42]. 

#### 4.2.6. Determination of the Antibacterial and Antifungal Effects of the GO Nanoemulsions 

On tryptic soy broth media, bacteria strains used in applications were reactivated from lyophilized stocks. The activated strains were dispersed on tryptic soy agar plates, and 100 µL of the GO nanoemulsion was added to the discs or wells to load them. According to Abdel Razek et al. [11], the diffusion assay was used to measure the antibacterial activity of the nanoemulsions. For each strain, the effect of the applied emulsion on the inhibition was measured as the apparent zone diameter (mm); the greater the effectiveness of the apparent zone diameter, the greater the influence of the concentration. The disk and well diffusion assays were achieved using applied extracts to determine the antifungal effect against the fungal strains cultured on Czapek–Dox agar media.

The examined strains of fungi were activated preliminary on Czapek–Dox media. About 100uL of each fungal strain’s spore suspension (105 CFU/mL) was poured into applied plates containing well-diffusion filled with film composites. The inhibition impact was determined as a clear zone diameter around each well, utilizing the same conditions and methodology described by Abu-sree et al. [16]. 

#### 4.2.7. Determination of Minimal Inhibition Concentration of the GO Nanoemulsions 

The minimal inhibitory and fungicidal concentration (MIC) value is determined as the lowest concentration of extract that did not give any visible bacterial growth. Each assay was carried out in triplicate. The MIC test quantified the antimicrobial activity of the extract, and it was conducted as the method described by Sakanaka et al. [43]. The minimal fungicidal inhibition (MFC) was determined for the emulsion against toxigenic fungal strains, as defined by Picman et al. [44]. Nystatin can be used as a positive control. The regression equation will calculate the concentration required to give 50% inhibition of hyphen growth IC50. 

#### 4.2.8. Anti-Aflatoxin Simulation Experiment Using Edible Film Application

A strain of *A. flavus* ITEM 698, a high aflatoxins producer, was utilized to evaluate the anti-aflatoxigenic impact of two emulsion solutions (film base (1) and film base (2)). Conidial suspension of fungi preparation was collected from 7-day-old cultures by pouring a sterile 0.01 % aqueous Tween 80 solution onto the culture plates and scraping the plate surface with a bent glass rod to facilitate conidia release. Cultures of fungal isolates (5 days old) cultivated on potato dextrose agar (PDA) slants at 28 °C were used to prepare the spore suspension. The conidia-containing inoculum was transferred to a new tube, and its optical density was adjusted to 0.5 McFarland standards. The final inoculum concentration was chosen at 1.2 × 10^3^ to 1.3 × 10^3^ colony-forming units per mL (CFU/mL). Using a Burker–Turk counting chamber, the number of conidia was adjusted using a Heamocytometer slide.

#### 4.2.9. Determination of Aflatoxin Reduction in Simulated Liquid Media

Through the filtrated media resulting from the previous step, aflatoxins (AFs) were extracted and estimated in the growth media of the *A. flavus* strain. The AOAC-approved extraction technique was used to evaluate aflatoxins [45]. The culture broth (10 mL) was mixed twice with 10 mL chloroform, then separated using a separating funnel following shaking vigorously for 10 min. The chloroform was evaporated under nitrogen; the lower phase was treated over anhydrous extra-pure sodium sulfate. To dissolve the aflatoxin dry film, HPLC-grade acetonitrile was utilized. One milliliter of the solution was mixed with 10 mL of distilled water before being deposited on an Afla-test immunological affinity column and rinsed twice with 10 mL distilled water (flow rate: 6 mL/min). To elute AFs, 2 mL of the acetonitrile (flow rate: 0.3 mL/min) was utilized. The quantitative analyses were performed using a pre-calibrated fluorometer (VICAM Series 4EX Fluorometer, Watertown, MA, USA; LOD 1.0 g/L).

#### 4.2.10. Molecular Docking

The crystal structures of polyketide synthase (A0A1R3RGK0) and cytochrome P450 monooxygenase (A0A1R3RGJ7) were obtained from the UniProt database (https://www.uniprot.org/, accessed on 3 February 2022), followed by removing the co-crystallized ligands and ions and then protonation using the Pymol software (Version 2.5.1). The MMFF94 force field was used in Avogadro Software (Version 1.2.0) to optimize the 3D structures of the ligands, which were downloaded from the PubChem database (http://pubchem.ncbi.nlm.nih.gov, accessed on 2 July 2022) [46]. A web-based software called CB-DOCK2, accessed on 2 July 2022 (http://clab.labshare.cn/cb-dock/php/), was used to execute blind docking. After submission, CB-Dock2 verifies the input files and uses OpenBabel and MGLTools to convert them to pdbqt formatted files. AutoDock Vina receives each center, size, and pdbqt file for docking. The protein’s cavities are then predicted by CB-Dock, which determines the centers and dimensions of the top N (n = 5 by default) cavities. After N rounds of computing, the final findings are shown [47]. The benchmarks carried out by Liu et al. [47] demonstrated that CB-Dock2 beat other blind docking tools in terms of their success rates for top-ranking poses, whose root mean squared deviation (RMSD) was within 2 Å from the position in the X-ray crystal structure. Utilizing the Discovery Studio program (Version 21.1.0.20298), the interface and visualization profiles for the best-docked complexes were created [48].

#### 4.2.11. Statistical Analysis

The Excel program used one-way ANOVA and the least significant difference (*p* = 0.05) test to compare the statistical data analysis. Mean values and standard deviations from at least three replicates were used to express the results. The significant differences between the extract concentrations were evaluated to distinguish between the individual mean significant changes at the 0.05% level.

## 5. Conclusions

Ginger oil was effective as an anti-aflatoxigenic material with antifungal impact. Loading ginger oil in composites used for coating nuts is a successful technique against microbial contamination. The FB2-GO coating material possessed an antifungal effect that was more efficient against toxigenic fungal strains. Applying the ginger oil to the composite coating material showed microbial activity differentiated according to the composite formation. However, the nanoemulsion characterization was also changed due to the building-up materials of the composite. The better ability manifested the efficiency of the coating-composite material for the mycelia reduction in *Aspergillus* strains and their ability to produce aflatoxins in a simulated liquid media. These fundings recommend their application to preserve nuts during the post-harvest stage. The efficacy of ginger essential oil, particularly as a loaded oil on the composites, was explained throughout its impact on aflatoxin-related enzymes of fungal cell growth using the simulated media. Using the molecular docking application, active components of *α*-bisabolene, *ar*-curcumene, and *α*-zingiberene presented a piece of evidence for aflatoxin reduction. Docking activity illustrated the interaction with the main enzymes responsible for the aflatoxin formation. 

## Figures and Tables

**Table 1:** The particle size, zeta potential, and poly dispersing index of the microemulsion prepared using garlic extracts.

Sample	Droplet Size (nm)	Zeta Potential (mV)	PDI	% ES	Acidity (as g Citric/L)
(FB1)	289.77 ± 15.34 ^a^	+31.54 ± 1.08 ^a^	0.37 ± 0.05 ^a^	87.34 ± 1.88 ^a^	0.84 ± 0.05 ^a^
(FB2)	126.54 ± 8.41 ^b^	+46.25 ± 1.05 ^b^	0.26 ± 0.07 ^b^	95.64 ± 2.54 ^b^	0.21 ± 0.07 ^b^

Data were expressed as the means ± SD (where n = 3); the means with different superscript letters (a, b) were significant differences for each column. FB1—coated emulsion composite consisted of maltodextrin, CMC, Arabic gum, and Guar gum. FB2—coated emulsion composite consisted of sodium alginate and whey protein; PDI: poly dispersing index; ES: emulsion stability.

**Table 2:** The identification of the volatile constituents of the volatile ginger oil using GC-MS.

S/N	Compound	RI ^a^	LRI ^b^	*Area*%			Identification Method ^c^
				GO	FB1-GO	FB2-GO	
**1**	α-Pinene	937	939	1.43	1.23	0.91	RI, MS, STD
**2**	Camphene	943	946	1.25	-	-	RI, MS, STD
**3**	α-Myrcene	986	991	0.09	-	-	RI, MS, STD
**4**	α-Terpinene	1009	1014	0.01	-	-	RI, MS, STD
**5**	Limonene	1020	1024	1.42	-	-	RI, MS, STD
**6**	β-Phellandrene	1023	1025	1.52	-	-	RI, MS, STD
**7**	1,8-Cineole	1028	1026	1.29	-	-	RI, MS, STD
**8**	Linalool	1100	1095	1.62	-	0.21	RI, MS, STD
**9**	Borneol	1160	1165	1.34	2.14	-	RI, MS, STD
**10**	α-Terpineol	1189	1186	1.79	-	-	RI, MS, STD
**11**	Neral	1231	1235	5.21	1.1	-	RI, MS, STD
**12**	Geranial	1244	1246	10.87	2.96	1.51	RI, MS, STD
**13**	α-Copaene	1375	1374	2.53	-	-	RI, MS
**14**	β-Elemene	1387	1389	2.23	1.08	1.31	RI, MS
**15**	cis-β-Farnesene	1443	1442	1.78	1.47	2.07	RI, MS
**16**	trans-β-Farnesene	1458	1456	2.98	2.93	3.37	RI, MS
**17**	*γ*-Muurolene	1479	1478	5.21	6.22	4.9	RI, MS
**18**	*ar*-Curcumene	1483	1480	5.96	14.52	16.23	RI, MS, STD
**19**	*α*-Zingiberene	1495	1493	31.8	41.99	39.96	RI, MS, STD
**20**	*β*-Bisabolene	1506	1505	8.19	9.35	9.49	RI, MS. STD
**21**	*α*-Bisabolene	1510	1507	5.21	14.96	18.43	RI, MS
**22**	Z-Nerolidol	1530	1531	2.98	-	-	RI, MS
**23**	*α*-Eudesmol	1650	1653	0.53	-	-	RI, MS
**24**	Cedrenol acetate	1738	1742	0.89	-	-	RI, MS
**25**	(*2E*, *6E*)-Farnesol	1747	1743	1.04	-	-	RI, MS
	Total	-	-	99.17	99.95	98.39	-

**RI ^a^**—retention indices calculated on DB-5 column using alkanes standards. **LRI ^b^**—retention indices according to literature. **^c^** Confirmed by comparison with the retention indices, the mass spectrum of the authentic compounds, and the NIST mass spectra library data.

**Table 3:** The antibacterial and anti-aflatoxigenic properties of the nanoemulsion composites containing ginger oil.

*Microbiological Strains*	GO	FB1	FB2	FB1-GO	FB2-GO
***Bacterial strains***					
*Listeria monocytogenes* ATCC 15313	10.22 ± 2.51	6.21 ± 0.84	5.02 ± 0.67	14.24 ± 1.02	12.34 ± 1.13
*Bacillus cereus* *NRRL 569*	10.27 ± 3.14	6.52 ± 0.71	5.66 ± 0.63	14.69 ± 1.37	12.66 ± 1.41
*Klebsiella aerogenes* *ATCC 13048*	9.66 ± 1.31	6.11 ± 0.51	4.74 ± 0.44	13.81 ± 1.31	12.14 ± 0.66
*Pseudomonas aeruginosa* *ATCC 9027*	9.05 ± 2.08	6.08 ± 0.41	4.28 ± 0.54	13.69 ± 1.05	12.08 ± 1.24
***Toxigenic Aspergillus strains***					
*Aspergillus flavus* *ITEM 698*	13.05 ± 1.02	5.75 ± 1.05	7.37 ± 1.31	17.34 ± 1.56	25.88 ± 1.67
*A. parasiticus* *ITEM 11*	12.33 ± 2.44	5.89 ± 1.11	7.71 ± 1.55	16.37 ± 1.88	24.41 ± 1.92
*A. nomius* *NRRL 13137*	14.77 ± 2.58	6.25 ± 1.36	8.44 ± 1.61	20.08 ± 1.69	28.24 ± 1.51

The results were represented as the mean ± SEM (standard error means; n = 3). The results were expressed as the inhibition zone diameter (clear zone surrounding the growth) measured in millimeters (mm). FB1—coated emulsion composite consisted of maltodextrin, CMC, Arabic gum, and Guar gum. FB2—coated emulsion composite consisting of sodium alginate, whey protein, and GO—ginger oil.

**Table 4:** The degradation of mycelial growth by film composites in simulated liquid media.

Mycelial Weight (g)	*A. flavus* Weight (g)	Inhibition (%)	*A. parasiticus* Weight (g)	Inhibition (%)
Control	3.941 ± 0.054	--	4.088 ± 0.076	--
FB1	3.573 ± 0.087	9.33	3.977 ± 0.274	2.72
FB2	2.821 ± 0.144	28.42	3.116 ± 0.174	23.77
GO	2.17 ± 0.231	44.93	2.21 ± 0.258	45.93
FB1+GO	1.194 ± 0.051	70.79	1.374 ± 0.041	65.13
FB2+GO	0.306 ± 0.134	92.51	0.596 ± 0.277	84.87

The results were represented as the mean ± SEM (standard error means; n = 3). The results were expressed as mycelia weight measured in gram (g). Inhibition (%)—represents the reduction ratio compared to the control that occurred in the composites treatment (10 µL/mL) in liquid media. FB1—coated emulsion composite consisted of maltodextrin, CMC, Arabic gum, and Guar gum. FB2—coated emulsion composite consisting of sodium alginate, whey protein, and GO—ginger oil.

## Data Availability

All data related to the present study are presented in the manuscript.
